# Dietary Egg White Hydrolysate Prevents Male Reproductive Dysfunction after Long-Term Exposure to Aluminum in Rats

**DOI:** 10.3390/metabo12121188

**Published:** 2022-11-28

**Authors:** Caroline Silveira Martinez, Jose Antonio Uranga-Ocio, Franck Maciel Peçanha, Dalton Valentim Vassallo, Christopher Exley, Marta Miguel-Castro, Giulia Alessandra Wiggers

**Affiliations:** 1 Graduate Program in Biochemistry and Multicentric Graduate Program in Physiological Sciences, Universidade Federal do Pampa, BR 472-Km 592, P.O. Box 118, Uruguaiana 97500-970, Rio Grande do Sul, Brazil; 2 William Harvey Research Institute, Heart Centre, Barts and The London School of Medicine and Dentistry, Queen Mary University of London, Charterhouse Square, London EC1M 6BQ, UK; 3 Department of Ciencias Básicas de la Salud, Universidad Rey Juan Carlos, Avda. de Atenas s/n, 28032 Alcorcón, Spain; 4 Departments of Physiological Sciences, Universidade Federal do Espírito Santo, Av. Marechal Campos 1468, Vitória 29040-090, Espírito Santo, Brazil; 5 The Birchall Centre, Lennard-Jones Laboratories, Keele University, Staffordshire ST5 5BG, UK; 6 Bioactivity and Food Analysis Laboratory, Instituto de Investigación en Ciencias de la Alimentación, Nicolás Cabrera, 9, Campus Universitario de Cantoblanco, 28049 Madrid, Spain

**Keywords:** environmental contaminant, heavy metal, functional food, bioactive peptides

## Abstract

Aluminum (Al) is a non-essential metal omnipresent in human life and is considered an environmental toxicant. Al increases reactive oxygen production and triggers immune responses, contributing to chronic systemic inflammation development. Here, we have tested whether an egg white hydrolysate (EWH) with potential bioactive properties can protect against changes in reproductive function in rats exposed to long-term Al dietary levels at high and low doses. Male Wistar rats received orally: low aluminum level group—AlCl_3_ at 8.3 mg/kg b.w. for 60 days with or without EWH (1 g/kg/day); high aluminum level group—AlCl_3_ at 100 mg/kg b.w. for 42 days with or without EWH (1 g/kg/day). The co-administration of EWH prevented the increased Al deposition surrounding the germinative cells, reducing inflammation and oxidative stress in the reproductive organs. Furthermore, the daily supplementation with EWH maintained sperm production and sperm quality similar to those found in control animals, even after Al exposure at a high dietary contamination level. Altogether, our results suggest that EWH could be used as a protective agent against impairment in the reproductive system produced after long-term exposure to Al at low or high human dietary levels.

## 1. Introduction

Human exposure to aluminum (Al) is unavoidable during the entire lifespan, and the numerous ways we are exposed to Al ensures that it will be notably more so in the future [1]. However, the mechanisms of absorption, metabolism, deposition, and excretion of Al through the human body still need to be well elucidated in part due to the absence of biological function of this non-essential metal [2]. Furthermore, the consequences for human health from exposure to Al continue to be reported in the scientific literature [3,4], but they are still mostly unknown compared to other human contaminants [5,6].

Human exposure to Al occurs through dietary and non-dietary sources. Al salts are added to commercially available foods and are used to treat drinking water and the packaging and storage of various food products [7]. Among the non-dietary sources, Al is found in pharmaceuticals, cosmetics, and deodorants and is used as an adjuvant to improve vaccines’ immune response [8]. Upon reaching a body burden threshold, Al shows a non-linear dose-response toxicity, as evidenced by recent research [9,10].

Al exposure triggers hematological, renal, cardiovascular, endocrine, reproductive, and auto-immune disorders [11,12,13,14]. Al^+3^ appears to disproportionally increase reactive oxygen production through Fenton reaction and trigger immune responses which may contribute to chronic systemic inflammation [14,15]. Al has been considered a trigger of the onset, progression, and outcomes of Alzheimer’s disease (AD) [16,17,18]. Although the relationship to metal exposure has been documented to be strongly related to damage to the nervous system and associated with neurodegenerative diseases, it seems not restricted to the central nervous system.

An elevated concentration of Al has been found in human sperm in correlation with a reduction in sperm quality [13]. Experimentally, rodents exposed to considerably high Al levels develop testicular abnormalities, endocrine disruption, and impaired reproduction. Based on this, the effects of burgeoning human Al exposure on female and male reproductive systems have been gaining attention during the last decades, mainly due to the increased infertility rate among couples and the exponential decrease in human sperm quality [19,20]. Recently, we have investigated the effects of Al exposure at human dietary levels, and we observed that it could impair spermatogenesis and sperm quality [21]. Moreover, it has been described that reproductive dysfunction can be achieved even at low doses of Al [21,22]. 

Considering the growing and inevitable human exposure to Al, strategies are necessary to counteract or reduce its impact on human and reproductive health. Our group has tested the bioactive properties of an egg white hydrolysate (EWH) treated for 8 h with pepsin against numerous cardiometabolic complications and metal exposure consequences [23,24,25]. These multi-functional peptides have potent anti-inflammatory and antioxidant capacities [23,24,25], which could be of value in preventing or minimizing the consequences of Al exposure. In addition, in a model of Al exposure at human dietary levels, EWH prevented memory impairment and vascular dysfunction after long-term exposure [26,27].

Here, we report our investigation of the anti-inflammatory and antioxidant regulatory effects of EWH as protection against reproductive dysfunction induced by low and high levels of dietary Al in Wistar rats. This study highlights for the first time that dietary supplementation with EWH peptides protects against reduced sperm quality after Al exposure at low and high dietary Al levels.

## 2. Materials and Methods 

### 2.1. Preparation of EWH

EWH was prepared from pasteurized egg white, treated with pepsin for 8 h, centrifuged, and the supernatants were frozen and lyophilized as previously described [24]. For treatment, lyophilized EWH was resuspended in water and administered by gavage. In addition, some bioactive peptide sequences previously identified (FRADHPFL, RADHPFL, YAEERYPIL, YRGGLEPINF, ESIINF, RDILNQ, IVF, YQIGL, and SALAM) were checked by HPLC-MS/MS [23,28] prior to administration.

### 2.2. Animal Treatment 

Male Wistar rats (90 days old, 360 ± 10.5 g) were obtained from the Charles River Animal Laboratory (Barcelona, Spain). Animals were housed under standard conditions (constant room temperature, humidity, and 12:12 h light-dark) with water and food available ad libitum (commercial diet A04, SAFE, Augy, France). The experimental protocol presented in the current study was performed following the Brazilian Societies of Experimental Biology and the European Legislation on Care and Use of Experimental Animals (EU Directive 2010/63/EU; R.D. 53/2013). This work was approved by Brazilian and Spanish Ethics Committees on Animal Use (CEUA, Universidade Federal do Pampa, Brazil—028/2014; Universidad Rey Juan Carlos, Spain—39/2014). 

Male Wistar rats were randomly distributed into two main groups as described [10,26] and received orally and daily:

Low aluminum level group—rats were divided into four subgroups (*n* = 8) (a–d) and received for 60 days: (a) Control—ultrapure water as liquid intake in the bottle, continuously throughout the day + ultrapure water by gavage once a day (Milli-Q, Merck Millipore Corporation, Billerica, MA, USA); (b) Al 8.3—Al at 8.3 mg/kg b.w. in ultrapure water as liquid intake in the bottle + ultrapure water by gavage once a day, representing human dietary Al intake [10]; (c) Hydrolysate (EWH)—ultrapure water as liquid intake in the bottle + EWH at 1 g/kg/day by gavage once a day [29]; (d) Al 8.3 + EWH—Al at 8.3 mg/kg b.w. in ultrapure water as liquid intake + EWH at 1 g/kg/day by gavage.

High aluminum level group—rats were divided into four subgroups (*n* = 8) (e–h) and received daily for 42 days: (e) Control—ultrapure water by oral gavage once a day; (f) Al 100—Al at 100 mg/kg b.w. by oral gavage once a day, dose considered as a super-dietary Al intake [30]; (g) Hydrolysate (EWH) —ultrapure water and EWH at 1 g/kg/day by gavage once a day; (h) Al 100 + EWH—Al at 100 mg/kg b.w. by gavage + EWH at 1 g/kg/day. All animals received ultrapure water as liquid intake. 

The stock solutions of Al (AlCl_3_.6 H_2_O at 0.034 M, for the low aluminum level group, 8.3 mg/kg/b.w. versus 0.331 M, for the high aluminum level group, 100 mg/kg/b.w.) were prepared in ultrapure water. 

In the low aluminum level group, rats were exposed to Al in their drinking water for 60 days to better represent human dietary Al exposure [10]. In the high aluminum level group, animals were treated for 42 days by oral gavage and received tap water as drinking water to induce Al toxicity representing a high level of human exposure to Al [30].

The amount of Al contained in the animals’ chow and drinking water could increase the amount of rats’ Al intake per day. Therefore, the Al content of rat chow was previously determined by TH GFAAS (Transversely Heated Graphite Furnace Atomic Absorption Spectrometry) [10]. The total Al content in rats’ feed (86.03 ± 4.76 μg Al/g/feed) was considered to calculate the daily Al exposure. Moreover, this protocol used special care to prevent inclusion of extraneous Al: only plastic materials were used, all laboratory ware (pipette tips, volumetric flasks) were immersed for at least 48 h in a 10% (*v*/*v*) HNO_3_/ethanol solution and washed with Milli-Q purified water before use. To avoid contamination from the air, all steps in the reagents’ preparation were carried out in a class 100 clean bench. The Al solutions were prepared with purified water and the control groups also received purified water.

### 2.3. Sperm Quality Analysis

#### 2.3.1. Daily Sperm Production per Testis, Sperm Number and Transit Time in Epididymis

Homogenization-resistant testicular spermatids (stage 19 of spermiogenesis) and sperm in the caput/corpus epididymis and cauda epididymis were counted as described [31] with modifications [21]. The daily sperm production was calculated by dividing the number of spermatids at stage 19 by 6.1, corresponding to the number of days these spermatids are present in the seminiferous epithelium. Sperm transit time calculation through the epididymis considered the number of sperm in each portion of the epididymis divided by the daily sperm production.

#### 2.3.2. Sperm Morphology

Sperm morphology was analyzed according to Filler’s [32] criteria with modifications [21]. For that, sperm were obtained from the vas deferens and stored in 10% formal-saline. On the day of the analysis, smears were prepared on histological slides, and 200 spermatozoa per animal were evaluated under 400X magnification (Binocular, Olympus CX31, Tokyo, Japan). Morphological abnormalities were classified into the head (amorphous, banana and detached head), and tail morphology (bent and broken tail).

#### 2.3.3. Sperm Motility

Sperm were removed from the vas deferens by internal rising with 1 mL of Human Tubular Fluid (DMPBS-Nutricell-SP, São José do Rio Preto, Brazil) pre-warmed to 34 °C. Then, a 10 μL aliquot was transferred to a histological slide, and the sperm motility was analyzed [21]. A total of 100 spermatozoa per animal were analyzed by light microscopy (20× magnification, Binocular, Olympus CX31, Tokyo, Japan), and the motility was classified as type A: motile with progressive movement, type B: motile without progressive movement and type C: immotile. Sperm motility was expressed as percent of total sperm.

### 2.4. Biochemical Assay

Biochemical studies of oxidative stress biomarkers were performed in plasma and reproductive tissues. At the end of the treatments, animals were euthanized by decapitation, the testis, epididymis and prostate were removed, and quickly dissected and homogenized in 50 mM Tris HCl, pH 7.4, (1/10, *w*/*v*). Afterward, tissue samples and blood were centrifuged at 2400× *g* for 10 min at 4 °C, and the resulting supernatant fraction and plasma were frozen at −80 °C for further assay. 

#### 2.4.1. Reactive Species Levels

The levels of reactive species (RS) in testis, epididymis, and prostate were determined by a spectrofluorometric method, as described [33] with previous modifications [21]. The RS levels (reactive oxygen species and reactive nitrogen species) are based on fluorescent 2′,7′-dichlorofluorescein (DCF) formed by the hydrolysis of 2′,7′-dichlorofluorescein diacetate (DCHF-DA) added to the medium. DCF fluorescence intensity is proportional to the amount of RS formed being recorded at 520 nm (with 480 nm excitation using SpectraMax M5, Molecular Devices, San Jose, CA, USA) for 60 min at 15 min intervals and expressed as fluorescence units.

#### 2.4.2. Lipid Peroxidation

Lipid peroxidation levels in testis, epididymis, and prostate were determined as malondialdehyde (MDA) levels according to the colorimetric method of Ohkawa [34], with modifications [21]. The color reaction was measured at 532 nm against blanks (SpectraMax M5, Molecular Devices, San Jose, CA, USA), and the results were expressed as nmol MDA/mg protein.

#### 2.4.3. Ferric Reducing/Antioxidant Power (FRAP) Assay

The total antioxidant capacity in the testis, epididymis, and prostate was measured by Ferric Reducing/Antioxidant Power (FRAP) assay according to Benzie & Strain [35], with modifications [21]. The absorbance was read at 593 nm (SpectraMax M5, Molecular Devices, CA, USA), normalized using a dose-response curve of trolox (50–1000 μM—vitamin E analog) and expressed regarding trolox equivalents. 

### 2.5. Testis and Epididymis Histology

The testis and epididymis histological studies were carried out as previously described [21]. Briefly, epididymis was fixed in 10% formaldehyde and testis in Bouin solution for two days, washed, embedded in paraffin, and sectioned at 5 µm. Tissues were stained with hematoxylin/eosin and analyzed using a Zeiss Axioskop 2 microscope (Zeiss, Jena, Germany). Morphometric parameters such as the seminiferous epithelium thickness (μm), the average number of empty seminiferous tubules per field, and the average number of efferent ducts per epididymal field were investigated. The images were analyzed in AxioVision 4.6 software (Zeiss, Jena, Germany). The epithelium thickness was measured in all tubules in testis sections, regardless of stages of spermatogenesis. In addition, 10 random fields per animal and eight animals per experimental group were analyzed under 20× magnification. 

### 2.6. Testis Immunohistochemistry 

The presence of inflammation in the testis was verified by immunohistochemistry on paraffin-embedded tissues as previously described [21]. Briefly, de-paraffined slides were incubated with hydrogen peroxide, blocked with fetal bovine serum, and incubated overnight at 4 °C with a monoclonal antibody against the pro-inflammatory M2 macrophage-associated antigen CD163 (1:100, Santa Cruz Biotechnology, Inc., Santa Cruz, CA, USA). The peroxidase-based kit Masvision (Master Diagnostica, Granada, Spain) was used as chromogen; slides were counterstained with hematoxylin and the presence and number of macrophages were quantified (20× magnification).

### 2.7. Aluminum Content in Testis and Epididymis

The Al content in the testis and epididymis was determined by TH GFAAS (Transversely Heated Graphite Furnace Atomic Absorption Spectrometry) using an established method [36] described elsewhere [21]. Results were expressed in μg Al/g tissue dry massmass as the arithmetic means of triplicate analysis.

### 2.8. Lumogallion Staining

The presence of Al in tissues was further confirmed in Bouin and formalin-fixed testis and epididymis by the lumogallion staining method [37,38]. Briefly, tissues were rehydrated and placed for 45 min into either 1 mM lumogallion (TCI Europe *n*.V., Zwijndrecht, Belgium) buffered in 50 mM PIPES, pH 7.4 or in PIPES-buffer alone for auto-fluorescence analyses. After several washes with PIPES-buffer, slides were rinsed in ultrapure water, mounted using an aqueous mounting medium and stored at 4 °C overnight before imaging. Tissues were observed using a Zeiss Axioskop 2 microscope. 

### 2.9. Statistical Analysis

Parametric results are expressed as mean ± SEM (standard error of the mean), nonparametric results as median, first quartile and third quartile (Q1–Q3). Data were analyzed using GraphPad Prism 8 (GraphPad Software, Inc., San Diego, CA, USA) and two-way ANOVA was performed; when ANOVA showed a significant treatment effect, Bonferroni’s post hoc test was used to compare individual means. The analysis of sperm motility was carried out by the Kruskal–Wallis test, followed by Dunn’s multiple comparisons test. Values were considered statistically different when *p* < 0.05.

## 3. Results

### 3.1. Body and Organs Mass, Daily Sperm Production, Sperm Number, Morphology, and Motility

Neither Al nor EWH treatments modified the rats’ body mass gain (Table 1 and Table 2). However, the reduction of 66% in the prostate mass after Al exposure at the highest dose was totally prevented by the co-treatment with pepsin-EWH (Table 2).

The concomitant administration of EWH was able to prevent the reduction in sperm quality after Al exposure at both low and high dietary Al levels. Furthermore, rats exposed to either Al dose and co-treated with EWH showed an increase in daily sperm production, in sperm number and sperm content in the testis and epididymis, similar to the control group (Table 3 and Table 4).

**Figure 1:**
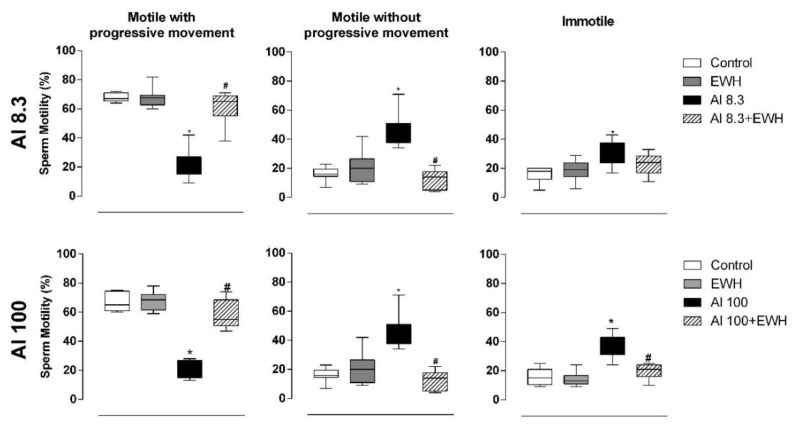
Effect of EWH on sperm motility of rats exposed to AlCl_3_ at 8.3 or 100 mg/kg b.w. per day. The motility is classified as: motile with progressive movement, motile without progressive movement and immotile. Data are expressed as median (Q1–Q3), *n* = 8, * *p* < 0.05 compared with their corresponding controls, # *p* < 0.05 compared with AlCl_3_ group (Kruskal–Wallis followed by Dunn’s test).

The EWH treatment protected rats against increased head and tail sperm abnormalities. While Al, independent of the doses, increased sperm abnormalities by 480%, both groups of rats exposed to Al and co-treated with EWH showed increases of only 150% in sperm abnormalities compared to respective controls (Table 5 and Table 6). Regarding sperm motility, Al groups showed a significant decrease while the animals cotreated with pepsin-EWH were able to preserve the percentage of motile sperm with progressive movement similar to control groups (EWH-Al groups 57.8%—controls 67.3% vs. Al groups 21.7%—Figure 1).

### 3.2. Reactive Species, Lipid Peroxidation, and Total Antioxidant Capacity 

**Figure 2:**
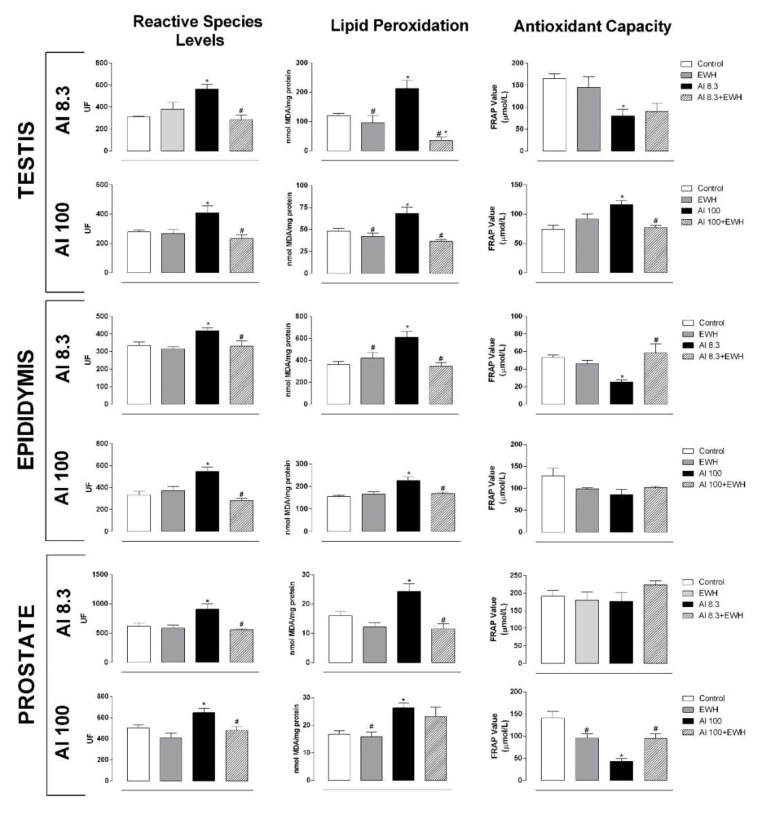
Effect of EWH on biochemical analysis of oxidative stress in rats exposed to AlCl_3_ at 8.3 or 100 mg/kg b.w. per day. Values of reactive oxygen and nitrogen species, lipid peroxidation and total antioxidant capacity on testis, epididymis, and prostate. UF: units of fluorescence, MDA: malondialdehyde, FRAP: ferric reducing / antioxidant power. Data are expressed as mean ± SEM, *n* = 8, * *p* < 0.05 compared with their corresponding controls, # *p* < 0.05 compared with AlCl_3_ group (two-way ANOVA and Bonferroni as post-hoc test).

Al exposure to the low (8.3 mg) or high dose (100 mg) induced oxidative stress in reproductive organs, impairing the antioxidant capacity and raising RS levels by 128% and 130% in the testis, 121% and 129% in the epididymis, and by 129% and 120% in the prostate, respectively, and compared to respective controls (Figure 2). However, both co-treatments with EWH were able to prevent the increased oxidative stress (Al + EWH—RS levels in percentages related to controls, testis: 71% and 69%, epididymis: 104% and 69%, prostate: 90% and 99%), keeping RS levels similar to those found in control animals. Similarly, the pepsin-EWH prevented the increase in lipid peroxidation in both Al-exposed animals, showing levels similar to control and EWH groups (Figure 2). 

### 3.3. Testis and Epididymis Histology

**Figure 3:**
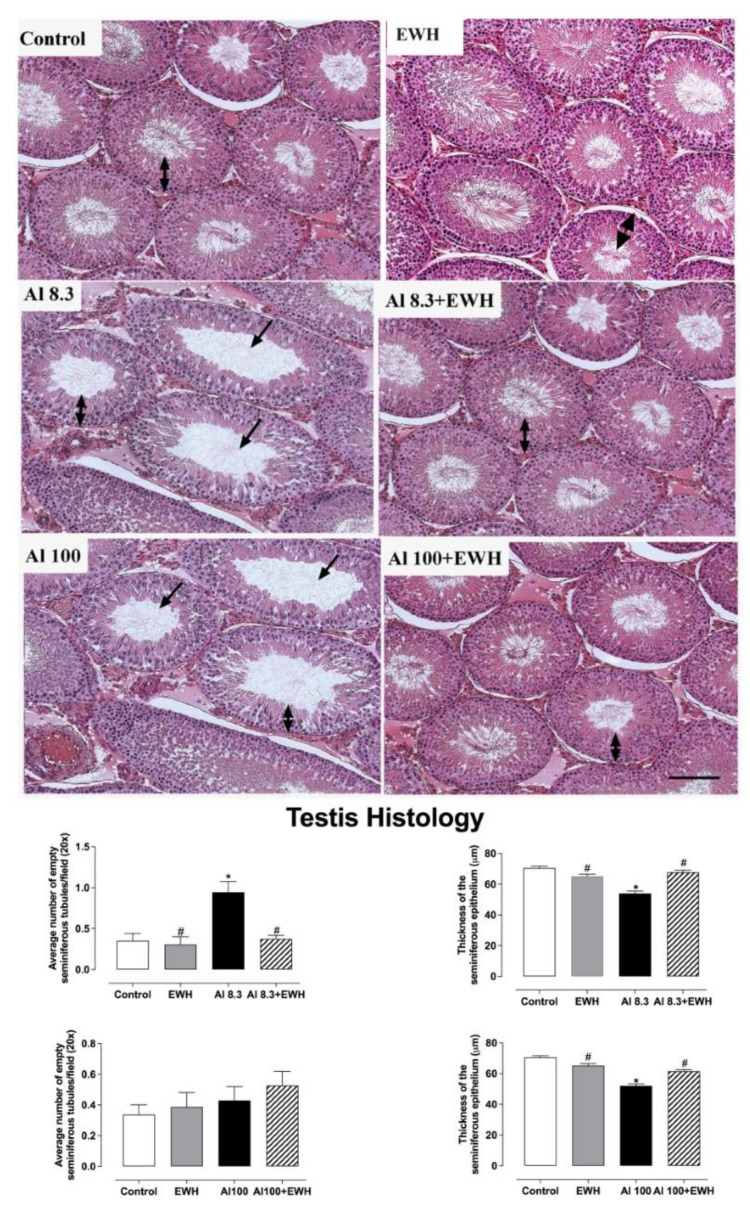
Effect of EWH on testis histopathology of rats exposed to AlCl_3_ at 8.3 and 100 mg/kg b.w. per day. Testis sections of Al-treated rats show a reduction of spermatozoa in the lumen of the seminiferous tubules (arrows) and reduced thickness of the seminiferous epithelium (µm—double arrows). Rats exposed to Al and co-treated with EWH show histology analysis similar to control and EWH-treated groups. Left graphs: Average number of empty seminiferous tubules per field (20×) in absolute numerical values. Right graphs: Thickness of the seminiferous epithelium (µm). Scale bar: 50 µm. Data are expressed as mean ± SEM, *n* = 6, * *p* < 0.05 compared with their corresponding controls, # *p* < 0.05 compared with AlCl_3_ group (two-way ANOVA and Bonferroni as post-hoc test).

**Figure 4:**
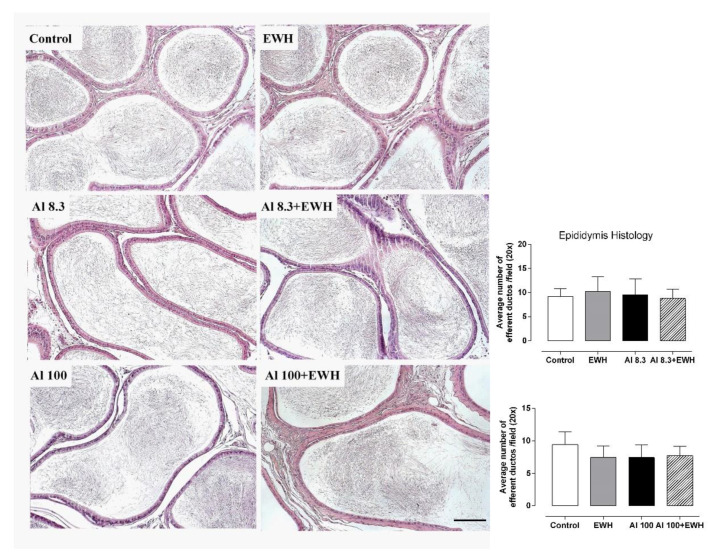
Effect of EWH on epididymis histopathology of rats exposed to AlCl_3_ at 8.3 and 100 mg/kg b.w. *per day*. Epididymis sections show normal histology for all groups. Scale bar: 50 µm. Graphs: Average number of empty efferent ducts per field (20×). Data are expressed as mean ± SEM, *n* = 6 (two-way ANOVA and Bonferroni as post-hoc test).

Al impaired testis histology but showed no effects on epididymis histoarchitecture (Figure 3 and Figure 4). The supplementation with pepsin-EWH was able to prevent the reduction in sperm number and in the thickness of the seminiferous tubules evoked by either Al dose (Figure 3). The epididymis histology showed no differences between the epididymis structure from control, EWH and Al-groups, showing a similar number of empty efferent ducts (means varying from 7 to 10 per field—Figure 4). 

### 3.4. Testis Immunohistochemistry 

**Figure 5:**
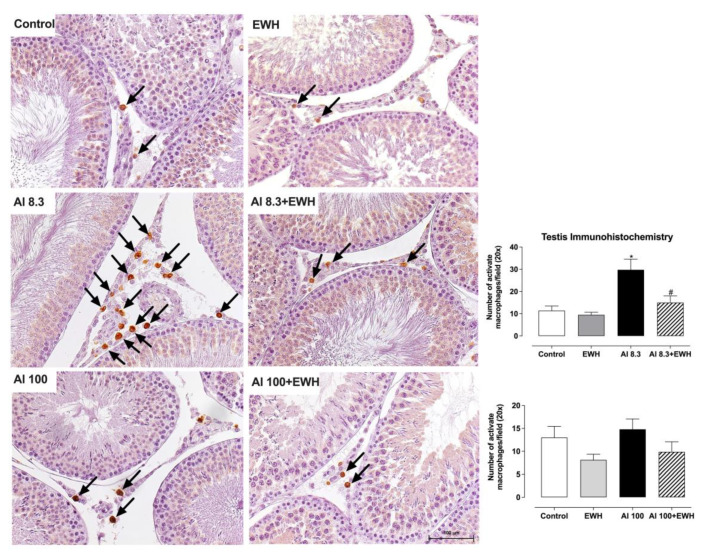
Effect of EWH on testis immunohistochemistry of rats exposed to AlCl_3_ at 8.3 or 100 mg/kg b.w. per day. Testis sections showing activated macrophages (arrows) detected by immunohistochemistry. Scale bars: 100 µm. Graphs: Average numbers of activated macrophages per field (objective 20×). Data are expressed as mean ± SEM, *n* = 6, * *p* < 0.05 compared with their corresponding controls, # *p* < 0.05 compared with AlCl_3_ group (two-way ANOVA and Bonferroni as post-hoc test).

The oral uptake of EWH prevented the development of inflammation in the testis after exposure to Al at 8.3 mg/kg bw, the human-relevant dietary level, reducing the number of activated macrophages by half when compared to Al (Al 8.3 group, ranging from 21 to 40; Al 8.3 + EWH group, ranging from 8 to 24 per field). The number of activated macrophages did not increase after Al exposure at the highest dose (Al 100 ranging from 10 to 20), and this is similar among groups (Figure 5).

### 3.5. Aluminum Content and Lumogallion Staining in Testis

**Figure 6:**
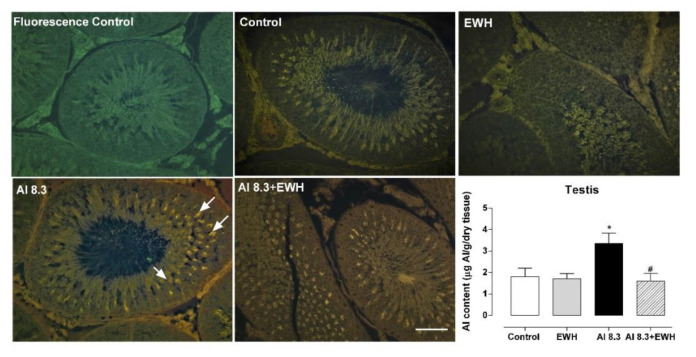
Effects of EWH on aluminum content in testis of rats exposed to AlCl_3_ at 8.3 b.w. per day. Representative testis sections showing lumogallion fluorescence for presence of aluminum (arrows), auto-fluorescence and nonspecific fluorescence in control and EWH treated rats. Scale bar: 50 µm. Graph: Al content (μg Al/g tissue dry mass) in testis determined by TH GFAAS (Transversely Heated Graphite Furnace Atomic Absorption Spectrometry). Data are expressed as mean ± SEM, *n* = 5, * *p* < 0.05 compared with the corresponding control, # *p* < 0.05 compared with AlCl3 group (two-way ANOVA and Bonferroni as post-hoc test).

Dietary EWH supplementation was able to prevent the Al deposition in testis evoked by Al exposure at 8.3. The lumogallion bright orange fluorescence highlights the specific presence of Al mainly located in the apical area of the seminiferous epithelium in Al treated groups, which was less specific in animals co-treated with EWH (Figure 6). 

## 4. Discussion

Results of our study highlight the capacity of dietary supplementation with bioactive peptides from an antioxidant and anti-inflammatory EWH to prevent or reduce the deleterious effects of Al exposure on the male reproductive system. The harmful effects of Al exposure have been demonstrated to be equivalent to low or high human dietary levels on the reproductive system, but not following a logical dose-response behavior. Some complications associated with Al exposure are not dependent on the concentration of the metal, but rather on the stimulus (pathways) that this metal could be triggering. In previous works, Al accumulated in testis induced macrophage activation and increased RS production in reproductive organs. These actions impair sperm characteristics needed for its viability, such as progressive motility and normal morphology, and sperm production in the testis with consequent sperm count reduction in the testis and epididymis of male rats [21]. 

We previously reported the in vitro antioxidant properties of EWH [24,39]. After in vivo studies, we determined that the consumption of EWH prevented inflammation and oxidative stress in different models of cardiometabolic disease and metal exposures [25,29,40]. In the present study, the oral uptake of 1 g/kg b.w. of pepsin-EWH prevented the reproductive dysfunction induced by dietary Al exposure at low and high intakes. In addition, the EWH prevented Al deposition within the testis, controlled inflammation, oxidative stress, and impaired spermatogenesis and sperm quality observed after Al exposure. 

Exposure to Al in the general population, including susceptible groups such as children or pregnant women, is continuously increasing [41,42]. However, very few studies have addressed the consequences of Al exposure on human health. Our study supports the hypothesis that less is not precisely safer in the case of Al exposure and that small amounts of Al are sufficient to trigger adverse effects. We know that animal model findings cannot be directly extrapolated to human exposures; however, outcomes due to the constancy observed in the results obtained with different animal models of Al exposure [9,43] raise a concern about exposure to Al in the general population, which could have worse consequences in a more susceptible population. Supporting this concern, a recent metallome study in pregnant women revealed that Al might play a role in the onset of central nervous system malformations. Specifically, a direct relationship was found between maternal serum concentration of Al and congenital defects of the central nervous system [44]. 

In the global scenario of declining sperm quality and increasing male-female couples suffering from infertility, it is essential to understand the causalities and contributors to this phenomenon [20,45]. Male factors account for 50% of worldwide infertility cases, and accumulating evidence supports the link between exposure to contaminants and lifestyles to male reproductive function [46,47,48]. 

The presence of Al in seminal human plasma has been investigated in the last decades, and a high concentration of Al seems to be related to low sperm quality, specifically reduced motility and sperm count among individuals showing some rate of reproductive dysfunction [13,49,50]. Al exposure in vitro seems to have a higher impact on human sperm motility and MDA production when compared with other common contaminants such as cadmium or lead [51]. Rodent models of exposure to Al support its impact on male reproductive health [52,53]. Al at doses ranging from 25 mg/kg b.w./day to 256.72 mg/kg b.w./day and different routes of exposure seem to disrupt the neuroendocrine system, reducing testosterone, luteinizing and follicular stimulating hormone, act locally in testis and epididymis inducing germ cell degeneration, atrophy and apoptosis and increasing sperm abnormalities in rodents [53,54,55].

The doses of Al of 8.3 mg/kg b.w./day, previously developed by our group, might be considered equivalent to a normal dietary intake of Al by human [10]. This dose was calculated according to a published protocol described by [56] and translated to an animal dose, based on a body surface area normalization method [57]. The high dose of Al exposure at 100 mg/kg b.w./day, is a protocol known to promote cognitive impairment in rats [30]. 

Herein, we have found that dietary supplementation with a pepsin-EWH was able to prevent the deleterious effects on reproductive organs after Al exposure at either low or high human-relevant doses. Specifically, in the animal model, we have shown that Al at low or high human dietary level impairs sperm function and spermatogenesis and that a pepsin-EWH added to the animal diet as a functional food ingredient prevented the effects of Al in testis, epididymis, and prostate, maintaining the sperm production per testis and the sperm quality similar to sperm quality found in the control animals. Moreover, EWH supplementation prevented Al deposition in the testis, the increased RS and lipid peroxidation levels, the macrophage differentiation and infiltration. Testes and spermatozoa are very susceptible to oxidative damage due to abundant polyunsaturated fatty acids (PUFAs) in the sperm membrane and their limited antioxidant capacity, which appears to be a standard feature of male infertility [58,59]. Our results suggest an ability of EWH to counteract the pro-oxidant effects of Al^3+^, which could be up-regulating the antioxidant capacity, then reducing oxidative damage within reproductive tissues. 

Indeed, the hydrolysis of the EWH with pepsin for 8 h releases peptides with several bioactive properties [23]. Among them, antioxidant properties of EWH have been demonstrated in vitro [23,29], reaching a peroxyl radical-scavenging activity of 574 μmol Trolox/g protein, which seems to be related to the presence of peptide sequences with Tyr at N-terminal residue [23]. Furthermore, the antioxidant activity of EWH has been proven in animal models [24,25,26,27,60]. Therefore, the antioxidant capacity could be related to its protective and beneficial effects against the harmful consequences of Al exposure on the reproductive system. 

Previous studies of our group have suggested the functional effect of EWH to protect against neurological, cardiovascular, and reproductive dysfunctions induced by heavy metals at low and high levels [25,26,27,60,61]. The daily consumption of pepsin-EWH also modulates inflammation by decreasing plasma levels of tumor necrosis factor-alpha and cyclooxygenase-2 activation in animals [24,27]. In the present study, the EWH also prevented the macrophage activation evoked by Al exposure in testis. Therefore, the protective role of daily EWH supplementation seems to be due to controlled oxidative stress and balanced inflammation status. 

Considering the antioxidant properties attributed to EWH and that EWH and Al have been administered separately, it is most likely that chelation occurs after both components are metabolized, during their passage through the digestive tract, or after in a localized manner in one of the target tissues. It has been described that food components, including food proteins, can act as potent metal chelators, thereby reducing metal concentration in blood and other organs [62]. Moreover, the production of hydrolysates derived from food proteins containing peptides with tyrosine and phenylalanine residues that have chelating capacity has been described [63]. In addition, some authors have suggested that sulfur-rich compounds, as is the case with some proteins and peptides, could reduce the absorption or reabsorption of toxic metals, and enhance natural detoxification pathways, since the efficiency of a chelating compound is related to its sulfur composition, a molecule that has a high affinity for toxic metals [64]. In fact, egg white is a food matrix largely composed of sulfur-containing proteins [65]. Therefore, it is conceivable that the antioxidant peptides contained in the egg white hydrolysate used in this work could act as chelators of the metal, thus reducing its absorption and enhancing its natural excretion pathways.

Another underlying mechanism that could be indirectly involved in metal chelation could be derived from the properties of EWH to increase the antioxidant defense of the organism and specifically the levels of reduced glutathione in liver. One of the best-defined mechanisms on the metal detoxification process by chelation is through reduced glutathione [66]. This molecule can form complexes with different metals. It has been shown that metals can form stable complexes when combined with the sulfhydryl groups of the reduced glutathione molecule in a 1:2 ratio (metal ion: reduced glutathione) [67]. In previous studies an increase in the levels of this molecule was observed in EWH-treated animals exposed to a toxic metal [68], and it is possible that this mechanism may also be related with an increase in chelation capacity.

We must emphasize that humans are exposed to Al daily, which means that Al chronically intoxicates most humans. Contrarily, no drugs or chelators are clinically approved to remove Al from the body [69]. In this context, combined strategies aiming to increase the removal of Al from the human body and to prevent the effects of the remaining Al could be an alternative to reduce the impact of the overall chronic Al intoxication. Drinking silicon-rich mineral water (30 mg/L, 1 L/ day) appears to facilitate the excretion of Al from the body and improve cognitive function in Alzheimer’s patients [70]. In the reproductive system, the natural active product found in crucifers, such as broccoli sprouts and sulforaphane, showed the potential to lower AlCl_3_-induced testicular toxicity in rats [71]. Furthermore, several different sources of antioxidant substances such as vitamin C [72], selenium-rich yeast [73], Citrullus lanatus seed ethanol extract [74], tyrosyl (an antioxidant present in olive oil) [75], and curcumin [76] show protective effects against reproductive Al toxicity probably due to their antioxidant and antiapoptotic properties. Therefore, our results suggest that oral supplementation with pepsin-EWH at 1 g/kg b.w./day could prevent the harmful effects of Al´s increased human body burden. These data, combined with previous research, are encouraging and highlight the health benefits of dietary supplementation with pepsin-EWH, which could be used as a functional food ingredient and introduced into the human diet to help prevent the deleterious outcomes induced by long-term exposure to Al. However, further studies are necessary to better understand the underlying mechanisms of EWH and its health benefits.

## 5. Conclusions

Considering the actual circumstances and future prevision about the increasing human exposure to Al, there is a real need for treatments or solutions to diminish the impact of the “aluminum age” in our and the next generations. Al deposits in the testis induce oxidative stress and inflammation, which are partially prevented by the co-treatment with EWH, reflecting the normal spermatogenesis and sperm viability found in the control rats. Our study shows for the first time that the multi-functional EWH-derived peptides could be used as a natural strategy against Al reproductive toxicity. A daily intake of EWH counteracts the harmful effects of Al exposure on spermatogenesis and sperm quality, such as reduced progressive motility and increased tail and head sperm abnormalities. Although our results with rodent models cannot be directly extrapolated to humans and must be taken with precaution, they suggest a promising role for EWH to protect against Al-associated male reproductive dysfunction. 

## Figures and Tables

**Table 1:** Effect of EWH on body mass (g), absolute (g or mg) and relative (g/100 g or mg/100 g) mass of reproductive organs of male rats exposed to AlCl_3_ at 8.3 mg/kg for 60 days.

Parameters	Experimental Groups			
	Control (*n* = 8)	Al 8.3 (*n* = 8)	EWH (*n* = 8)	Al 8.3 + EWH (*n* = 8)
Initial body mass (g)	346 ± 12.05	330 ± 8.77	348 ± 7.30	328 ± 10.65
Final body mass (g)	460 ± 15.70	436 ± 6.44	453 ± 12.47	454 ± 7.03
Testis (g)	1.82 ± 0.04	1.75 ± 0.04	1.77 ± 0.03	1.80 ± 0.04
Testis (g/100 g)	0.40 ± 0.01	0.40 ± 0.01	0.39 ± 0.01	0.39 ± 0.01
Epididymis (mg)	565 ± 25.57	498 ± 10.44	559 ± 17.46	547 ± 21.06
Epididymis (mg/100 g)	125 ± 7.81	114 ± 3.78	124 ± 6.90	119 ± 4.85
Ventral prostate (mg)	445 ± 10.17	390 ± 8.55	444 ± 35.97	361 ± 30.25
Ventral prostate (mg/100 g)	94.24 ± 3.50	89.74 ± 3.21	95.19 ± 9.02	80.19 ± 7.78
Full seminal vesicle (g)	1.51 ± 0.11	1.45 ± 0.10	1.48 ± 0.08	1.30 ± 0.07
Full seminal vesicle (g/100 g)	0.34 ± 0.02	0.32 ± 0.02	0.32 ± 0.01	0.30 ± 0.01
Empty seminal vesicle (g)	0.66 ± 0.07	0.65 ± 0.08	0.65 ± 0.05	0.62 ± 0.08
Empty seminal vesicle (g/100 g)	0.15 ± 0.01	0.14 ± 0.02	0.14 ± 0.01	0.13 ± 0.02
Vesicular secretion (g)	0.85 ± 0.11	0.80 ± 0.07	0.83 ± 0.06	0.69 ± 0.09
Vas deferens (mg)	87.50 ± 6.33	120 ± 19.02	140 ± 19.02	113 ± 24.25
Vas deferens (mg/100 g)	20.35 ± 1.68	27.99 ± 4.05	32.26 ± 4.07	25.34 ± 5.21

Data are expressed as means ± SEM. The relative organ mass was calculated by use of the formula: organ mass/body mass × 100. Units: g: gram, mg: milligram; two-way ANOVA (*p* > 0.05).

**Table 2:** Effect of EWH on body mass (g), absolute (g or mg) and relative (g/100 g or mg/100 g) mass of reproductive organs of male rats exposed to AlCl_3_ at 100 mg/kg for 42 days.

Parameters	Experimental Groups			
	Control (*n* = 8)	Al 100 (*n* = 8)	EWH (*n* = 8)	Al 100 + EWH (*n* = 8)
Initial body mass (g)	365 ± 10.32	409 ± 9.57	385 ± 14.71	398 ± 8.64
Final body mass (g)	437 ± 7.81	452 ± 8.81	415 ± 11.73	445 ± 10.43
Testis (g)	1.69 ± 0.03	1.80 ± 0.04	1.71 ± 0.03	1.72 ± 0.04
Testis (g/100 g)	0.39 ± 0.01	0.39 ± 0.01	0.40 ± 0.01	0.39 ± 0.01
Epididymis (mg)	483 ± 22.13	502 ± 36.92	468 ± 13.30	511 ± 16.01
Epididymis (mg/100 g)	111 ± 6.08	110 ± 9.02	110.2 ± 4.69	117 ± 7.35
Ventral prostate (mg)	408 ± 28.66	312 ± 35.24	475 ± 30.77	440 ± 26.74
Ventral prostate (mg/100 g)	96.37 ± 8.81	64.44 ± 5.71 *	119 ± 6.01	101 ± 7.63 #
Full seminal vesicle (g)	1.33 ± 0.10	1.33 ± 0.10	1.53 ± 0.06	1.53 ± 0.14
Full seminal vesicle (g/100 g)	0.31 ± 0.03	0.31 ± 0.02	0.38 ± 0.01	0.36 ± 0.04
Empty seminal vesicle (g)	0.58 ± 0.08	0.53 ± 0.06	0.70 ± 0.07	0.72 ± 0.09
Empty seminal vesicle (g/100 g)	0.14 ± 0.02	0.12 ± 0.01	0.17 ± 0.01	0.17 ± 0.02
Vesicular secretion (g)	0.71 ± 0.09	0.80 ± 0.08	0.83 ± 0.07	0.81 ± 0.02
Vas deferens (mg)	96.75 ± 5.96	83.50 ± 1.19	90.60 ± 5.10	106 ± 10.77
Vas deferens (mg/100 g)	21.96 ± 1.36	17.68 ± 0.76	22.80 ± 1.13	23.60 ± 2.32

Data are expressed as means ± SEM. The relative organ mass was calculated by use of the formula: organ mass/body mass × 100. Units: g: gram, mg: milligram; * *p* < 0.05 vs. Control; # *p* < 0.05 vs. Al100 (two-way ANOVA, followed by Bonferroni test).

**Table 3:** Effect of EWH on sperm counts in testis and epididymis of rats exposed to AlCl_3_ at 8.3 mg/kg for 60 days.

Parameters		Experimental Groups		
Sperm count	Control (*n* = 8)	Al 8.3 (*n* = 8)	EWH (*n* = 8)	Al 8.3 + EWH (*n* = 8)
Testis				
Sperm number (×10^6^)	140 ± 6.12	70.4 ± 3.35 *	139 ± 6.61 #	130 ± 4 #
Sperm number (×10^6^/g)	101 ± 2.15	48.97 ± 3.55 *	97.51 ± 5.87 #	93.38 ± 4.53 #
DSP (×10^6^/testis/day)	22.93 ± 1	11.54 ± 0.55 *	22.86 ± 1.23 #	21.37 ± 0.65 #
DSPr (×10^6^/testis/day/g)	16.59 ± 0.35	8.02 ± 0.58 *	16.27 ± 1.04 #	15.29 ± 0.74 #
Epididymis				
Caput/Corpus				
Sperm number (×10^6^)	122 ± 5.73	93.17 ± 6.93 *	134 ± 5.84 #	127 ± 5.53 #
Sperm number (×10^6^/g)	364 ± 17.68	270 ± 17.33 *	400 ± 22 #	379 ± 12.38 #
Sperm transit time (days)	5.39 ± 0.34	8.19 ± 0.70 *	5.75 ± 0.16 #	5.95 ± 0.25 #
Cauda				
Sperm number (×10^6^)	169 ± 5.45	113 ± 5.04 *	163 ± 8.31 #	167 ± 10.57 #
Sperm number (×10^6^/g)	845 ± 24.95	610 ± 25.83 *	825 ± 48.83 #	831 ± 44.90 #
Sperm transit time (days)	7.44 ± 0.33	10.14 ± 0.91 *	7.01 ± 0.30 #	7.90 ± 0.61

Data are expressed as mean ± SEM. Units: g: gram, mg: milligram. * *p* < 0.05 vs. Control; # *p* < 0.05 vs. Al 8.3 (two-way ANOVA followed by Bonferroni).

**Table 4:** Effect of EWH on sperm counts in testis and epididymis of rats exposed to AlCl_3_ at 100 mg/kg for 42 days.

Parameters			Experimental Groups	
Sperm count	Control (*n* = 8)	Al 100 (*n* = 8)	EWH (*n* = 8)	Al 100 + EWH (*n* = 8)
Testis				
Sperm number (×10^6^)	128 ± 4.69	62.3 ± 6.75 *	135 ± 6.15 #	114 ± 5.26 #
Sperm number (×10^6^/g)	94.17 ± 6.84	42.59 ± 3.93 *	94.44 ± 6.28 #	84.56 ± 3.07 #
DSP (×10^6^/testis/day)	21.06 ± 0.76	10.21 ± 1.10 *	21.80 ± 1.02 #	18.73 ± 0.86 #
DSPr (×10^6^/testis/day/g)	15.44 ± 1.12	6.98 ± 0.64 *	15.51 ± 1.02 #	13.86 ± 0.50 #
Epididymis				
Caput/Corpus				
Sperm number (×10^6^)	132 ± 5.58	98.9 ± 4.94 *	140 ± 6.15 #	131 ± 5.24 #
Sperm number (×10^6^/g)	410 ± 16.44	271 ± 19.72 *	379 ± 21.40 #	392 ± 14.30 #
Sperm transit time (days)	6.32 ± 0.38	10.57 ± 1.3 *	6.23 ± 0.3 #	7.04 ± 0.36 #
Cauda				
Sperm number (×10^6^)	190 ± 8.02	114 ± 8.93 *	172 ± 6.83 #	160 ± 9.53 #
Sperm number (×10^6^/g)	905 ± 31.82	589 ± 18.63 *	855 ± 40.68 #	847 ± 18.71 #
Sperm transit time (days)	8.66 ± 0.31	11.52 ± 0.64 *	7.73 ± 0.26 #	8.59 ± 0.49 #

Data are expressed as mean ± SEM. Units: g: gram, mg: milligram. * *p* < 0.05 vs. Control; # *p* < 0.05 vs. Al 100 (two-way ANOVA followed by Bonferroni).

**Table 5:** Effect of EWH on sperm morphology of rats exposed to AlCl_3_ at 8.3 mg/kg for 60 days.

Parameters	Experimental Groups			
	Control (*n* = 8)	Al 8.3 (*n* = 8)	EWH (*n* = 8)	Al 8.3 + EWH (*n* = 8)
Normal	96 (94–97)	76 (67–80) *	92.5 (91.2–95.7) #	92 (89.5–93.2)
Head Abnormalities				
Amorphous	0 (0–0.5)	9 (2–19) *	1 (0.2–2.7)	1 (0–3)
Banana Head	0 (0–1)	4 (1–6) *	0.5 (0–1)	2 (0.7–2.2)
Detached Head	0 (0–1)	2 (0–6)	0 (0–1)	0.5 (0–6)
Total of Head Abnormalities	2 (0–3)	15 (9–26) *	3 (1.2–3.7) #	3.5 (2–5.2)
Tail Abnormalities				
Bent Tail	0 (0–0.5)	5 (2–6) *	0 (0–1) #	0.5 (0–2)
Broken Tail	1 (0.0–2.5)	0 (0–1)	0 (0–0)	0 (0–0.2)
Total of Tail Abnormalities	3 (1–4.5)	9 (6–13) *	4.5 (0.7–6)	4 (2.7–6.2)

Data are expressed as median (Q_1_–Q_3_) * *p* < 0.05 vs. Control; # *p* < 0.05 vs. Al 8.3 (Kruskal–Wallis followed by Dunn’s test).

**Table 6:** Effect of EWH on sperm morphology of rats exposed to AlCl_3_ at 100 mg/kg for 42 days.

Parameters	Experimental Groups			
	Control (*n* = 8)	Al100 (*n* = 8)	EWH (*n* = 8)	AL100 + EWH (*n* = 8)
Normal	96 (94–97)	76 (67–80) *	92.5 (91.2–95.7) #	92 (89.5–93.2) *
Head Abnormalities				
Amorphous	0 (0–0.5)	9 (2–19) *	1 (0.2–2.7)	0 (0–3)
Banana Head	0 (0–1)	4 (1–6) *	0.5 (0–1)	2 (0.7–2.2)
Detached Head	0 (0–1)	2 (0–6)	0 (0–1)	0.5 (0–1)
Total of Head Abnormalities	2 (0–3)	15 (9–26) *	3 (1.2–3.7) #	3.5 (2–5.2)
Tail Abnormalities				
Bent Tail	1 (0–3)	4 (2–5) *	4.5 (0.2–5.7)	2.5 (1–4.5) #
Broken Tail	1 (0–2.5)	0 (0–1)	0 (0–0)	0 (0–0.2)
Total of Tail Abnormalities	3 (1–4.5)	9 (6–13) *	4.5 (0.7–6)	4 (2.7–6.2)

Data are expressed as median (Q1–Q3) * *p* < 0.05 vs. Control; # *p* < 0.05 vs. Al 100 (Kruskal–Wallis followed by Dunn’s test).

## Data Availability

The data presented in this study are available upon request from the corresponding author. The authors confirm that all data underlying the findings are within the paper.
